# Establishment of a cloning-free CRISPR/Cas9 protocol to generate large deletions in the bovine MDBK cell line

**DOI:** 10.1007/s13353-024-00846-3

**Published:** 2024-02-28

**Authors:** Joanna Stojak, Dominique Rocha, Caroline Mörke, Christa Kühn, Veronique Blanquet, Hiroaki Taniguchi

**Affiliations:** 1 Department of Experimental Embryology, Institute of Genetics and Animal Biotechnology, Polish Academy of Sciences, Jastrzębiec, Poland; 2 INRAE, AgroParisTech, GABI, Université Paris-Saclay, 78350 Jouy-en-Josas, France; 3 Research Institute for Farm Animal Biology (FBN), Institute of Genome Biology, Wilhelm-Stahl-Allee 2, 18196 Dummerstorf, Germany; 4 Agricultural and Environmental Faculty, University Rostock, 18059 Rostock, Germany; 5 Present Address: Friedrich-Loeffler-Institut (FLI), 17493 Greifswald, Insel Riems Germany; 6 Faculté Des Sciences Et Techniques, University of Limoges, 123 Avenue Albert Thomas, 87060 Limoges, France; 7 African Genome Center, University Mohammed VI Polytechnic (UM6P), Lot 660, Hay Moulay Rachid, 43150 Ben Guerir, Morocco

**Keywords:** CRISPR/Cas9, MDBK, Deletion, Regulatory elements, Cattle

## Abstract

The CRISPR/Cas9 technique applied to modify the cattle genome has value in increasing animal health and welfare. Here, we established a simple, fast, and efficient cloning-free CRISPR/Cas9 protocol for large deletions of genomic loci in the frequently used model bovine MDBK cell line. The main advantages of our protocol are as follows: (i) pre-screening of the sgRNA efficiency with a fast and simple cleavage assay, (ii) reliable detection of genomic edits primarily by PCR and confirmed by DNA sequencing, and (iii) single cell sorting with FACS providing specific genetic information from modified cells of interest. Therefore, our method could be successfully applied in different studies, including functional validation of any genetic or regulatory elements.

**Supplementary Information:**

The online version contains supplementary material available at 10.1007/s13353-024-00846-3.

## Introduction

The CRISPR technique allowed genome editing on annotated genomes of farm animals (Perisse et al. 2021), including the modifications of the cattle genome. Genetic engineering provides, therefore, the opportunity to enhance the efficiency of food production (e.g. milk, meat; e.g. Jabbar et al. 2021) and to improve animal health and welfare (e.g. hornless, heat tolerant, resistant to diseases; e.g. Carlson et al. 2016; Gao et al. 2017). However, there is still room for improvements, such as simplifying the method, especially since previous studies have confirmed that the successful outcome of genome editing with plasmid-based methods might depend on cell type. For instance, transfection of the Madin-Darby Bovine Kidney (MDBK) cells (Madin and Darby 1958), adherent epithelial cells frequently used as a bovine in vitro model (e.g. Li et al. 2018; Han et al. 2021; Jiang et al. 2022), is challenging due to low success rates (Osorio and Bionaz 2017). Moreover, a plasmid-based system has its limitations, including difficulties in efficiently transferring the plasmid into the nucleus (Nakamura et al. 2019; de Oliveira et al. 2019). Additionally, the methodology used in plasmid-based systems prevents testing the efficiency of sgRNAs (single guide RNAs) before the experiment is started.

**Fig. 1:**
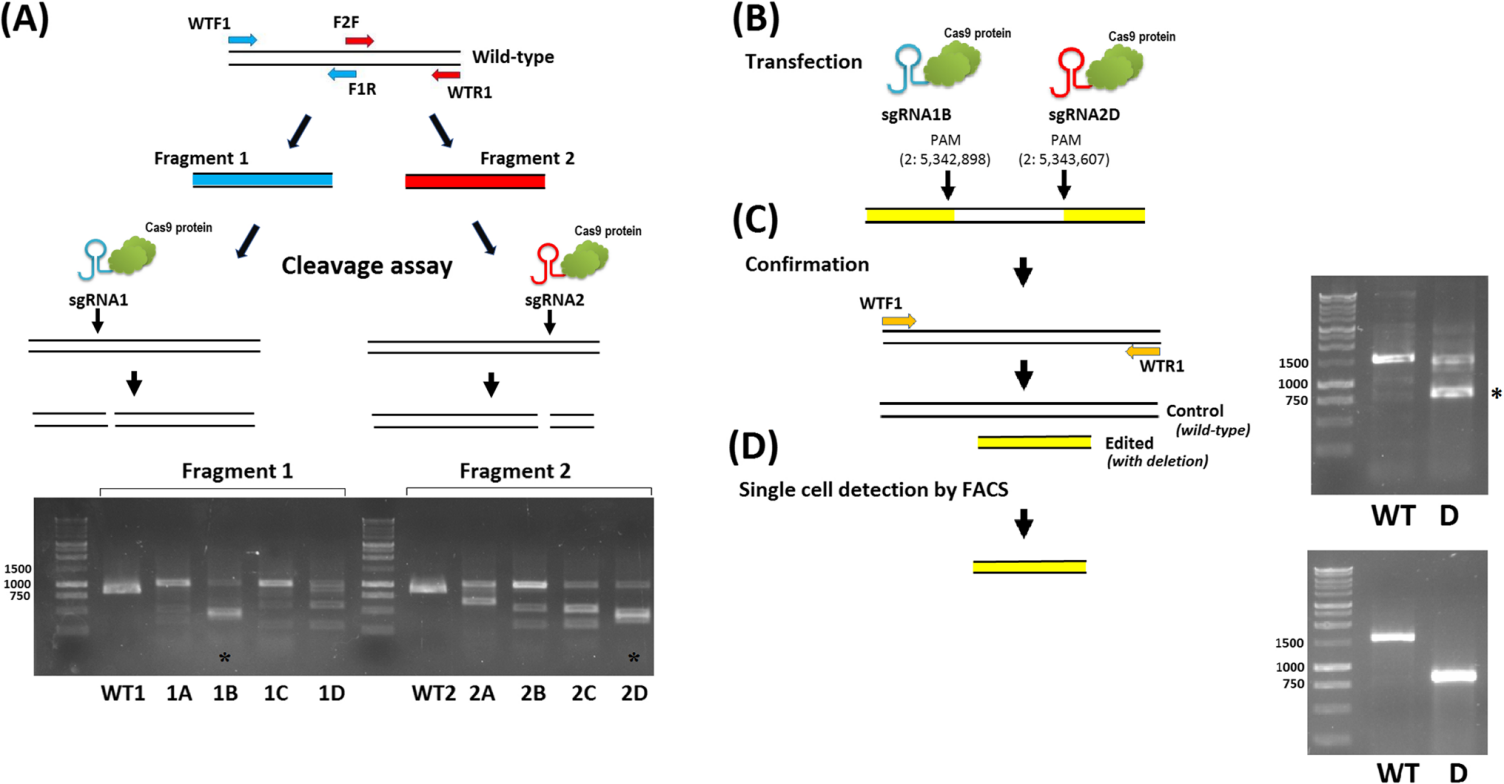
Cloning-free CRISPR/Cas9 approach for the generation of large genomic deletions using CRISPR/Cas9 and polymerase chain reaction (PCR) combined with gel electrophoresis. **A** Screening of sgRNA efficiency is performed by the cleavage assay on the region of interest amplified in two fragments. On the agarose gel, the overnight cleavage assay of the PCR products for fragment 1 and fragment 2 of the region of interest (Suppl. Table 2) using newly designed sgRNAs is shown. The PCR products before cleavage assay are marked by WT1 for fragment 1 and WT2 for fragment 2. The PCR products after the cleavage are marked by sgRNAs names (1A–1D for fragment 1 and 2A–2D for fragment 2). The most efficient gRNAs for each fragment (when less wild-type band is observed in the pattern after the cleavage and additional bands appeared after cleavage) for the further experiment are marked by an asterisk (*). **B** Transfection with ‘sandwich gRNA’ (combination of two selected sgRNAs). Below each sgRNA symbol, the PAM locations on chromosome 2 (BosTau9 version of the genome) are given. **C** Modifications in the genome after the transfection are detected primarily by PCR of the entire region of interest (different lengths of PCR products with and without deletion on agarose gel) and confirmed by DNA sequencing. The results of PCR amplification of the entire region of interest (using WTF1 and WTR1 primers) after the transfection of MDBK cells with the sandwich gRNAs are shown on the agarose gel. The PCR product for the wild-type (control) amplified using genomic DNA from MDBK cells before transfection is marked by ‘WT’. After transfection, the PCR product amplified using genomic DNA from MDBK cells where a possible deletion of the target element was detected in part of cells from the colony is marked by ‘D’. The band with deletion was marked by an asterisk (*). **D** FACS ensures accurate sorting of single cells (scattered by size) after transfection into 96-well plates, preventing the contamination by DNA from two single cells and providing specific genetic information from modified cell of interest only. On the agarose gel the wild-type (WT) and successful deletion (715 bp in length) of desired region D were shown

To solve these problems, we established a cloning-free CRISPR/Cas9 protocol to achieve large genomic deletions in MDBK cells. As a pilot study, we applied this protocol to delete a candidate regulatory element, identified by functional annotation of the cattle genome (ATAC peaks found in 15 cattle samples, representing all eight studied tissues; Kern et al. 2021). The cloning-free CRISPR approach, presented in this study, consists of a Cas9 endonuclease and a single-stranded guide RNA, which guides the Cas9 endonuclease to cleave both DNA strands of the target region in a sequence-specific manner. After the cleavage, the DNA is repaired by double-strand break repair mechanisms (Hille et al. 2018). Consequently, the CRISPR/Cas9 system introduces deletions or insertions, resulting in genome modifications. The efficiency of newly designed sgRNAs is confirmed before starting the experiment using the polymerase chain reaction (PCR) combined with gel electrophoresis (Fig. 1). The main advantages of our protocol are as follows: (i) gRNA is screened based on a fast and simple cleavage assay, (ii) modifications could be detected in a reliable manner by PCR and confirmed by DNA sequencing, and (iii) FACS ensures accurate sorting of cells after transfection into 96-well plates, preventing the contamination by DNA from two single cells and providing specific genetic information from modified cell of interest only. Therefore, the method proposed in this study can be successfully applied in different types of studies, including deletion of any genomic region in a fast, accurate, and cost-effective way.

## Materials and methods

Details on cell culture, selection and amplification of the targeted region, designing and in vitro screening of gRNAs, cell transfection, clone selection, and sequencing are provided in Supplementary Materials.

## Results and discussion

In this study, we present a protocol to obtain deletions of specific genomic regions in MDBK cells using CRISPR/Cas9 and PCR combined with gel electrophoresis as a quick and reliable approach to analyse the efficiency of designed sgRNAs.

Only a few studies have generated CRISPR-edited cell lines using MDBK cells. Chen et al. (2021) conducted transduction with lentiviral vectors encoding the sgRNAs under the control of a U6 promoter and Cas9 to produce novel MDBK *CD46* knockout clones, selected by interaction with the anti-bovine CD46 monoclonal antibody CA26. Other *CD46* knockout cells have been obtained by Szillat et al. (2020) using a CRISPR/Cas9 ribonucleoproteins (RNPs)-mediated approach. However, to our knowledge, a comprehensive protocol for CRISPR-mediated large genomic deletions using RNPs has not been reported.

Here, we described a swift and easy protocol for successfully designing and testing sgRNAs and the subsequent generation of a large deletion in the desired genome location. To choose and test the best-fitting crRNAs forming ‘sandwich gRNA’, we suggest designing and ordering several crRNAs in one batch. In our experiments, we used the combination of the two most efficient crRNAs (‘sandwich gRNA’) that cut the genomic DNA in two sites, at the beginning and the end of the desired region in the genome. In our method, several crRNAs were tested on MDBK DNA as a preliminary step using PCR and subsequent agarose gel visualization. The amplification of the wild-type region of interest was successful, and three amplicons were obtained—for fragment 1, fragment 2, and the entire wild-type region (Suppl. Table 1). The efficiency of the tested sgRNAs (1A–1D and 2A–2D) for fragment 1 and fragment 2 is presented in Fig. 1A. The crRNA-1B and crRNA-2D were chosen based on their cleavage efficiency (when, after cleavage, a less wild-type band is observed in the pattern together with additional bands present). After transfection of MDBK cells using the sandwich gRNAs (1B2D), genomic DNA was extracted, and the deletion was confirmed by PCR of the entire wild-type region, using WTF1 and WTR1 primers (Fig. 1B, C; Suppl. Table 1). The transfected cells with confirmed deletion (Fig. 1C) were separated by FACS into four 96-well plates and checked after 3 days.

In our protocol, we used 72-h incubation during the transfection process. Shorter incubation time (48 h) showed no significant difference in the transfection success (data not shown). The MDBK cells proliferated fast in our culture condition. Therefore, single-cell colony identification was possible after 3 days. In the case of other cell lines, it is important to adjust the protocol to the proliferation time.

The success rate (assessed by observation under the optical microscope) of monoclonal colony formation by FACS was 25.3% (97/384). Screening of all 97 potentially single-cell colonies, was performed after one additional week. Cells were trypsinized and diluted in 100 µl, of which 50 µl was used for PCR screening and visualization on agarose gel. Using this PCR screen, we can predict whether the obtained clone possesses homozygous or heterozygous deletions of the targeted region. Furthermore, this approach allows us to exclude the possible contamination of non-single cell-derived clones.

Only four clones with a clear single band of approximately 800 bp in length identified on the agarose gel (Fig. 1D), indicating successful deletion of the region of interest in a homozygote manner, were chosen for DNA sequencing. The DNA sequence was checked for all four clones and confirmed the targeted deletion (Suppl. Mats 1).

## Conclusions

Application of such easy-to-implement preliminary testing allows us to estimate the success rate of CRISPR/Cas9 modifications before they were even started in cell culture, saving time and reagents. Initial testing of crRNAs on DNA directly isolated from the cells on which work is planned (e.g. MDBK) provides confidence that the genome editing experiment will be efficient given that cell transfection is successful. It confirms that a large percentage of cells will undergo the CRISPR/Cas9 modifications. In spite of limited susceptibility to modifications, we were able to generate CRISPR-edited MDBK cell lines. However, various cell lines might differ significantly in susceptibility to genome modifications (Osorio and Bionaz 2017). Therefore, although our method worked for MDBK cells very efficiently, further tests should be performed with different cell lines.

### Supplementary Information

Below is the link to the electronic supplementary material.

Supplementary file1 (DOCX 31 KB)

## Data Availability

All data obtained in this study (protocol, DNA sequences) is fully presented in the article and supplementary materials.

## Declarations

### Disclaimer

The sole responsibility of this presentation lies with the authors. The Research Executive Agency is not responsible for any use that may be made of the information contained therein.
