# Supplementary material for: Establishment of a cloning-free CRISPR/Cas9 protocol to generate large deletions in the bovine MDBK cell line
Source: J Appl Genet. 2024 Feb 28;65(2):399–402. doi: 10.1007/s13353-024-00846-3 (PMC11003909; doi:10.1007/s13353-024-00846-3)
Supplement: Supplementary file 1 — Supplementary file1 (DOCX 31 KB) [file 13353_2024_846_MOESM1_ESM.docx]

**SUPPLEMENTARY MATERIALS**

**MATERIALS AND METHODS**

**Cell line**

The Madin-Darby bovine kidney (MDBK; Madin and Darby 1958) cell line is commercially available and cells used in this study were provided by prof. Ch. Kühn (FBN, Dummerstorf, Germany; see Demasius et al. 2013). Cells were cultured in Dulbecco’s modified Eagle’s medium (DMEM) high glucose (Biowest, France) that was supplemented with 10% fetal bovine serum (FSB) (EURx, Poland), 4,500 mg/l glucose, 100 µg/ml streptomycin, and 100 units/ml of penicillin. The cells were kept in a humidified incubator at 5% CO_2_ and 37°C.

**Choice and amplification of the targeted region**

The region on chromosome 2 (position between 5,342,432 and 5,344,050) in the cattle genome (version BosTau9) was used in this study. According to the Kern et al. (2021), this region contained ATAC peaks found in 15 different cattle samples (representing all 8 different studied tissues). This suggests the presence of a regulatory element.

Total genomic DNA was extracted from MDBK cells with the Genomic Mini kit (A&A Biotechnology, Poland), following the manufacturer’s instructions.

The PCR to amplify the entire region of interest in MDBK cells (1502 bp in length) was performed using the Terra PCR Direct Polymerase Mix (Takara Bio, Japan) in combination with newly designed specific primers (WTF1 and WTR1 for entire region of interest and primers WTF1, F1R, F2F and WTR1 for amplification of this region in two shorter, overlapping fragments (842 bp and 924 bp, respectively; Suppl. Table 1, Fig. 1A). The successful deletion of the targeted element is detected by PCR reaction using single set of primers (WTF1 and WTR1), resulting in PCR products of different length on agarose gels. In this study, the PCR product with amplified whole region of interest without deletion is defined as ‘wild-type region’. Two shorter fragments (and primers for their amplification, WTF1, F1R, F2F and WTR1) are used for sgRNAs testing only.

The following PCR conditions were used: 2 min at 98°C, then 36 cycles: 10 seconds at 98°C, 15 seconds at 62°C and 1 minute at 68°C, followed by incubation 5 minutes at 68°C and storage at 4°C. The negative controls without DNA template were used in each PCR reaction.

**Designing of gRNAs and *in vitro* screening**

Suitable CRISPR RNAs (crRNAs) were designed using the CHOPCHOP software (Labun et al. 2019) to introduce double-strand breaks upstream and downstream of the region of interest (Suppl. Table 2). The crRNAs (100 µM, without PAM sequence; NGG) were purchased from IDT (IA, USA). Annealing of the crRNAs with the trans-activating crRNA (1 µM, tracrRNA) to obtain sgRNAs was achieved by incubation of both components (0.6 µl each) in 58.8 µl of Duplex buffer (IDT, IA, USA) for 3 minutes at 95°C, followed by 30 minutes of cooling down to room temperature. The efficiency of the obtained sgRNAs was tested using a cleavage assay as follows: mix of 1 µl of sgRNA, 1 µM Alt-R HiFi Cas9 Nuclease (0.5 µl of *Streptococcus* *pyogenes* Cas9 protein was diluted in 30 µl of Opti-MEM, IDT, IA, USA;) and 15.5 µl of Opti-MEM (Thermo Fisher Scientific, MA, USA) was incubated for 2 minutes before adding of 2.5 µl of amplified PCR product, then was incubated overnight at 37°C. Four sgRNAs (1A-1D) were tested for the fragment 1 of the region of interest, and four other sgRNAs (2A-2D) were used to cleave fragment 2 (Suppl. Table 2). The size of PCR products before and after the cleavage assay were compared by gel electrophoresis on a 1.5% agarose gel in the presence of the Perfect Plus 1kB DNA ladder (EURx, Gdańsk, Poland) to confirm a successful cut in the genomic DNA. Two crRNAs (one of each cRNAs for fragment 1 and 2 of the analyzed region, 0.3 µl each) that worked best were chosen and combined with 0.6 µl of 1 µM tracrRNA (according to the protocol described above). Such mixture of two sgRNAs in 58.8 µl of Duplex buffer is defined in this study as ‘sandwich gRNA’ and was further used in the experiments leading to generation of CRISPR-edited MDBK cells with a targeted large deletion in the entire region of interest.

**Transfection of MDBK cells and clone selection**

The targeted deletion in the region of interest (expected size approximately 700 bp) in MDBK cells was done using a sandwich gRNA: a combination of two crRNAs, selected as described in the previous section. The transfection was done using 7.5 µl of sandwich gRNA mixture prepared in a previous section, 7.5 µl of 1µM Alt-R HiFi Cas9 Nuclease, 10 µl of Opti-MEM and 1.2 µl of Lipofectamine RNAiMAX (Thermo Fisher Scientific, MA, USA). The reaction mix was complemented to 50 µl by adding 23.8 µl of Opti-MEM and incubated for 15 minutes at room temperature before transferring the total mixture into single wells of 96-well plate. MDBK cells (wild-type, kept in T25 flasks until the experiment) were trypsinized and 2 x 10^4 cells were added in 100 µl of medium into a well of 96-well plate with the total mixture with Lipofectamine RNAiMAX described above. After 72h incubation, cells were detached from the well of 96-well plate by trypsinization and diluted in 100 µl, of which 50 µl was used for gDNA harvest to confirm editing via PCR (next day) and 50 µl was used for further proliferation (for 1 weeks in a single well of 96-well plate and then transferred into T25 flask for another week) and to obtain single cell colonies by FACS sorting into four 96-well plates. The FACS was performed by an external laboratory (Laboratory of Cytometry at Nencki Institute of Experimental Biology, Warsaw, Poland) using a BD FACSAria™ IIu cell sorter (scattering by cell size). Single cell colonies were incubated for 3 days (37°C, 5% CO_2_) and screened for the intended deletion. Screening PCR conditions were the same as in previous steps, using WTF1 and WTR1 primers for the entire wild-type region (Suppl. Table 1). The success of the CRISPR-mediated gene editing was checked by gel electrophoresis in 1.5% agarose gel.

**Sequencing**

The clones which had shorter PCR amplification products than the expected wild-type size, were sequenced by Eurofins Genomics (Köln, Germany) using Sanger sequencing. Sequencing results were analysed using the online tool Clustal Omega (https://www.ebi.ac.uk/Tools/msa/clustalo/).

**References**

1. Demasius W, Weikard R, Hadlich F, Müller KE, Kühn C (2013) Monitoring the immune response to vaccination with an inactivated vaccine associated to bovine neonatal pancytopenia by deep sequencing transcriptome analysis in cattle. Veterinary Research 44(1): 93. doi: 10.1186/1297-9716-44-93.

2. Labun K, Montague TG, Krause M, Torres Cleuren YN, Tjeldnes H, Valen E (2019) CHOPCHOP v3: expanding the CRISPR web toolbox beyond genome editing. Nucleic Acids Res 47(W1): W171-W174. doi: 10.1093/nar/gkz365

3. Madin SH, Darby NB Jr. (1958) Established kidney cell lines of normal adult bovine and ovine origin. Proc Soc Exp Biol Med 98(3): 574-6. doi: 10.3181/00379727-98-24111

4. Kern C, Wang Y, Xu X, Pan Z, Halstead M, Chanthavixay G, Saelao P, Waters S, Xiang R, Chamberlain A, Korf I, Delany ME, Cheng HH, Medrano JF, Van Eenennaam AL, Tuggle CK, Ernst C, Flicek P, Quon G, Ross P, Zhou H (2021) Functional annotations of three domestic animal genomes provide vital resources for comparative and agricultural research. Nature Communications 12(1): 1821. doi: 10.1038/s41467-021-22100-8.

**Supplementary Materials 1.** The alignment of DNA sequences of the region of interest obtained in this study for MDBK without modification (‘MDBK’) and MDBK single cell colonies with confirmed deletion (‘Deleted’) in comparison to original BosTau9 genome sequence. Congruent regions are marked by ‘*’. Differences between BosTau9 and wild-type MDBK are highlighted in yellow.

BosTau9 CTGCCTACTGGCAGCTCACAGGCTGGGCTATTGCTGCCTCATCTCTGCCCTGGGCACCAT 60

MDBK CTGCCTACTGGCAGCTCACAGGCTGGGCTATTGCTGCCTCATCTCTGCCCTGGGCACCAT 60

Deleted CTGCCTACTGGCAGCTCACAGGCTGGGCTATTGCTGCCTCATCTCTGCCCTGGGCACCAT 60

************************************************************

BosTau9 CTCTGAAAGTGGGGGCCTCACCGCTGATGGCTCATCGTGTGTAGCTCACCCAGGTTTGGT 120

MDBK CTCTGAAAGTGGGGGCCTCACCGCTGATGGCTCATCGTGTGTAGCTCACCCAGGTTTGGT 120

Deleted CTCTGAAAGTGGGGGCCTCACCGCTGATGGCTCATCGTGTGTAGCTCACCCAGGTTTGGT 120

************************************************************

BosTau9 TTTAAAATGAGGAGAAGGCCCCTCCTCCCACTCTGAGCACCTCTATCCCCTCATCTTGAG 180

MDBK TTTAAAATGAGGAGAAGGCCCCTCCTCCCACTCTGAGCACCTCTATCCCCTCATCTTGAG 180

Deleted TTTAAAATGAGGAGAAGGCCCCTCCTCCCACTCTGAGCACCTCTATCCCCTCATCTTGAG 180

************************************************************

BosTau9 TCTTCTCGGCAAAGCAGAGCTTCCCACCTTCCTGTGCCTCCCGCCGAGGCCCCAGCGACC 240

MDBK TCTTCTCGGCAAAGCAGAGCTTCCCACCTTCCTGTGCCTCCCGCCGAGGCCCCAGCGACC 240

Deleted TCTTCTCGGCAAAGCAGAGCTTCCCACCTTCCTGTGCCTCCCGCCGAGGCCCCAGCGACC 240

************************************************************

BosTau9 TGCCAGCTTTCTCCTTAAGTGTCTGTGGCAGTCTCCAGCTGCATCTTGTTGAGGACAAGC 300

MDBK TGCCAGCTTTCTCCTTAAGTGTCTGTGGCAGTCTCCAGCTGCATCTTGTTGAGGACAAGC 300

Deleted TGCCAGCTTTCTCCTTAAGTGTCTGTGGCAGTCTCCAGCTGCATCTTGTTGAGGACAAGC 300

************************************************************

BosTau9 ACAGCTGAGGGGTGAATTTCCTTCTTTCCTTGGCTCTCTCAGTCTTCACCACAGCCCCCA 360

MDBK ACAGCTGAGGGGTGAATTTCCTTCTTTCCTTGGCTCTCTCAGTCTTCACCACAGCCCCCA 360

Deleted ACAGCTGAGGGGTGAATTTCCTTCTTTCCTTGGCTCTCTCAGTCTTCACCACAGCCCCCA 360

************************************************************

BosTau9 GGATGCTCTTACTACCCCTCTTCGTTTGAGAAAACAGGCTCAGAACGGCATTGCTCACTC 420

MDBK GGATGCTCTTACTACCCCTCTTCGTTTGAGAAAACAGGCTCAGAACGGCATTGCTCACTC 420

Deleted GGATGCTCTTACTACCCCTCTTCGTTTGAGAAAACAGGCTCAGAACGCTATT-------- 412

****************************************************

BosTau9 AAAAATCATAATGAGGTGAGTCTCAGGTCACGTTGTCCGTGTAGTCCATATGGATTTATT 480

MDBK AAAAATCATAATGAGGTGAATCTCAGGTCACGTTGTCCGTGTAGTCCATACGGATTTATT 480

Deleted ------------------------------------------------------------ 412

BosTau9 GAGCCCCTACTGTGTGCTGTCTGCCTCCAAAACGCACTCTCTTTCTACACTTCCTGCTGC 540

MDBK GAGCCCCTACTGTGTGCTGTCTGCCTCCAAAACGCACACTCTTTCTACACTTCCTGCTGC 540

Deleted ------------------------------------------------------------ 412

BosTau9 CCCTCAAAGTGCTGCTCAGAAACAGGTCAGAGCAAGAGGCTTGGGGTGGGGATTGGGGGG 600

MDBK CCCTCAAAGTGCTGCTCAGAAACAGGTCAGAGCAAGAGGCTTGGGGTGGGGATTGGGGGG 600

Deleted ------------------------------------------------------------ 412

BosTau9 AGGTGGGTGCCAGTCCTGCAGATAGGACGGAACCATGCCTTGTCTCGCCACTAGATGGTG 660

MDBK AGGTGGGTGCCAGTCCTGCAGATAGGACGGAACCATGCCCTATCTCGCCACTAGATGGTG 660

Deleted ------------------------------------------------------------ 412

BosTau9 CTGCTGGCCCAGTCGTCCAGCGTTTGCTGCGGGAGCAGAGTCCAGCCCAGCAGCTCCTCT 720

MDBK CTGCTTGCCCGGTCGCCCAGCGTTTGCTGCGGGAGCAGAGTCCAGCCCAGCAGCTCCTCT 720

Deleted ------------------------------------------------------------ 412

BosTau9 GGGCCCATTTTTTCCTGAAAGCAGGGCCGCCCCAACCAAACAGGGAGAGCACTTTAAAGA 780

MDBK GGGCCCATTTTTTCCTGAAAGCAGGGCCGCCCCAACCAAACAGGGAGAGCACTTTAAAGA 780

Deleted ------------------------------------------------------------ 412

BosTau9 GGCCAGGAAGCCATTCCGAGGCAGTCTCACCCGTGGAAAGCTGGAGCCTGAGGGGCCTTT 840

MDBK GGCCAGGAAGCCATTCCGAGGCAGTCTCACCCGTGGAAAGCTGGAGCCTGAGGGGCCTTT 840

Deleted ------------------------------------------------------------ 412

BosTau9 GAGACTACCCAGCTCATGTCCTGCATGTTGGTTTCTTAGGAGTTGTGGCTGCTGAAGGCT 900

MDBK GAGACTGCCCAGCTCATGTCCTGCATGTTGGTTTCTTAGGAGTTGTGGCTGCAGAAGGCT 900

Deleted ------------------------------------------------------------ 412

BosTau9 CAGAAGGACCAAGAAGATGCTGGAGGGTAGACGCTAAGCCTGGCTGGCAGGGGGTTGATG 960

MDBK CAAAGGGACCAAGAAGATGCTGGAGGGTAGACGCTAAGCCTGGCTGGCAGGGGGTTGATG 960

Deleted ------------------------------------------------------------ 412

BosTau9 GGGAGAACCCTGGAAGCTATAGGACAGAGTAAAGGGACAAAGTAGGTGGTTAAATAGTCT 1020

MDBK GGGAGAACCCTGGAAGCTATAGGACAGAGTAAAGGGACAAAGTAGGTGGTTAAATAGTCT 1020

Deleted ------------------------------------------------------------ 412

BosTau9 AATACTTAATAGATTGTAAGTAAGTAGAGAAAATATGAGAGACCCCTGTAAATTAATGAT 1080

MDBK AATACTTAATAGATTGTAAGTAAGTAGAGAAAATATGAGAGACCCCTGTAAATTAATGAT 1080

Deleted ------------------------------------------------------------ 412

BosTau9 TACTCAAGAAAGCAGGTTTGTTTAAGCACAAAGCAGGCTTGCTTAAGCGAGAAAATCCTG 1140

MDBK TACTCAAGAAAGCAGGTTTGTTTAAGCACAAAGCAGCCTTGCTTAAGCGAGAAAATCCTG 1140

Deleted -----------------------------------------------CGAGAAAATCCTG 425

*************

BosTau9 CGACAGAAGCACTAGAAGAAAACAGCAAAGCCATGGTGGCCTGAGGACCACATCCTGCTA 1200

MDBK CGACAGAAGCACTAGAAGAAAACAGCAAAGCCATGGTGGCCTGAGGACCACATCCTGCTA 1200

Deleted CGACAGAAGCACTAGAAGAAAACAGCAAAGCCATGGTGGCCTGAGGACCACATCCTGCTA 485

************************************************************

**Supplementary Table 1.** Primers for the amplification of the region of interest, including melting temperature (Tm) and expected amplicon size.

| **Fragment** | **Primer name** | **Primers** | **Tm**  **(in °C)** | **Amplicon size** |
| --- | --- | --- | --- | --- |
| Entire wild-type region | WTF1  WTR1 | forward: 5’-AGTCTGTTCTTGGGCTTGCT-3’  reverse: 5’-AATTCGGTGAGAGGCGAAGT-3’ | 59.1  61.2 | 1502 bp |
| Fragment 1  (F1) | WTF1  F1R | forward: 5’-AGTCTGTTCTTGGGCTTGCT-3’  reverse: 5’- ATGGCTTCCTGGCCTCTTTA-3’ | 59.1  61.1 | 842 bp |
| Fragment 2  (F2) | F2F  WTR1 | forward: 5’- TCTCGCCACTAGATGGTGCT-3’  reverse: 5’- AATTCGGTGAGAGGCGAAGT -3’ | 61.0  61.2 | 924 bp |

**Supplementary Table 2.** The crRNAs used in this study.

| **Upstream of region of interest**  **(fragment 1)** | **Downstream of region of interest**  **(fragment 2)** |
| --- | --- |
| crRNA-1A:  5’-TGAGGACAAGCACAGCTGAG-3’ | crRNA-2A:  5’- CCATGCAGGGTCAAGAACTCTCC-3’ |
| crRNA-1B:  5’-TTGAGAAAACAGGCTCAGAA-3’ | crRNA-2B:  5’-AGCAAAGCCATGGTGGCCTG-3’ |
| crRNA-1C:  5’- GCTTTCTCCTTAAGTGTCTG-3’ | crRNA-2C:  5’-AGAAAACAGCAAAGCCATGG-3’ |
| crRNA-1D:  5’- GCCAGCTTTCTCCTTAAGTG-3’ | crRNA-2D:  5’-GGTTTGTTTAAGCACAAAGC-3’ |
